# Medical Society of Tennessee

**Published:** 1844-06

**Authors:** 


### MEDICAL SOCIETY OF TENNESSEE.

This society held its fifteenth annual meeting in Nashville on the 1st of May, and a copy of its proceedings is already on our table. The members who persevere in attending the meetings of this society, and labor to impart interest to them by communicating the results of their experience, deserve well of the public. They are fired by the proper zeal to raise the standing and contribute to the resources of their profession. We have repeatedly had occasion to applaud the spirit of this society. It has no toleration for quackery. A book was sent to it by Dr. Sappington, of Missouri, at its late meeting, which came in a rather questionable shape through a newspaper advertisement. Resolved by the Society, "that the book be returned to its author."

Interesting papers were read to the society which we expect to receive for insertion in our journal. Dr. Buchanan was re-elected President, and Dr. Thompson Vice President; Dr. C. K. Winston was elected Corresponding Secretary, Dr. Stout Recording Secretary, and Dr. R. Martin Treasurer of the Society. Many valuable specimens have been added, during the year, to the Society's Anatomical Museum. Y.

